# Noise and spectral stability of deep-UV gas-filled fiber-based supercontinuum sources driven by ultrafast mid-IR pulses

**DOI:** 10.1038/s41598-020-61847-w

**Published:** 2020-03-18

**Authors:** Abubakar I. Adamu, Md. Selim Habib, Callum R. Smith, J. Enrique Antonio Lopez, Peter Uhd Jepsen, Rodrigo Amezcua-Correa, Ole Bang, Christos Markos

**Affiliations:** 1 DTU Fotonik, Technical University of Denmark, Kgs. Lyngby, DK-2800 Denmark; 2 CREOL, The College of Optics and Photonics, University of Central Florida, Orlando, FL-32816 USA; 3 NKT Photonics, Blokken 84, Birkerød, 3460 Denmark; 4 NORBLIS IVS, Virumgade 35D, DK-2830 Virum, Denmark

**Keywords:** Supercontinuum generation, Ultrafast photonics

## Abstract

Deep-UV (DUV) supercontinuum (SC) sources based on gas-filled hollow-core fibers constitute perhaps the most viable solution towards ultrafast, compact, and tunable lasers in the UV spectral region, which can even also extend into the mid-infrared (IR). Noise and spectral stability of such broadband sources are key parameters that define their true potential and suitability towards real-world applications. In order to investigate the spectral stability and noise levels in these fiber-based DUV sources, we generate an SC spectrum that extends from 180 nm (through phase-matched dispersive waves - DWs) to 4 μm by pumping an argon-filled hollow-core anti-resonant fiber at a mid-IR wavelength of 2.45 μm. We characterize the long-term stability of the source over several days and the pulse-to-pulse relative intensity noise (RIN) of the DW at 275 nm. The results indicate no sign of spectral degradation over 110 hours, but the RIN of the DW pulses at 275 nm is found to be as high as 33.3%. Numerical simulations were carried out to investigate the spectral distribution of the RIN and the results confirm the experimental measurements and that the poor noise performance is due to the high RIN of the mid-IR pump laser, which was hitherto not considered in numerical modelling of these sources. The results presented herein provide an important step towards an understanding of the noise mechanism underlying such complex light-gas nonlinear interactions and demonstrate the need for pump laser stabilization.

## Introduction

Fiber-based SC sources are remarkably bright, spatially coherent light sources that can span from DUV to the mid-infrared (mid-IR) spectral region. DUV laser sources, in particular, have numerous important applications in the semiconductor industry^1^, such as in photolithography and chip inspection^2^, as well as in time-resolved spectroscopy^3^. These applications require a stable and low noise laser source^4^. Although the most stable laser sources are fiber-based^5^, many of these fiber lasers use solid-core silica fibers with extremely low loss in the near-IR region, but with extremely high attenuation in the UV and mid-IR regions, rendering them unsuitable for delivery of UV and mid-IR light. Alternatively, solid-core soft-glass fibers, such as ZBLAN and chalcogenide fibers, have been demonstrated to be suitable to provide a spectrum extending into the mid-IR^6,7^ and several commercial mid-IR SC sources are now available covering wavelengths up to about 4.9 µm. Single-wavelength mid-IR lasers are now available at around 2 µm (Thulium-doped silica fibers) and 3 µm (Er-doped ZBLAN fibers)^8^. However, solid-core silica fiber based UV laser sources are yet to be realized^9,10^. The main limitations of fused silica for UV sources are, multiphoton absorption^11^, radiation-induced photodarkening (also known as solarization) as well as significant material absorption^9^.

The fluoride glass ZBLAN has a short wavelength loss edge of about 190 nm and could therefore be used to transmit UV light. However, SC sources require a zero-dispersion wavelength (ZDW) close to the pump, which implies that the core of the ZBLAN fiber must be extremely small to support UV SC generation. Only one demonstration of a UV SC in a ZBLAN fiber has thus been made, in which a unique and never replicated ZBLAN Photonic Crystal Fiber (PCF) was fabricated with a core diameter of about 3 µm. This core diameter was still not small enough to match the dispersion requirements for SC generation, so the authors had to couple the light to the ~150 nm interstices between the holes in the PCF cladding structure to achieve a suitable ZDW that allowed the generation of an SC extending down to 200 nm^12^. The fabrication of ZBLAN PCFs with so small core sizes still remains a challenging task, therefore they are also considered not a viable route towards DUV SC sources.

Hollow-core photonic crystal fibers (HCPF), on the other hand, overcome the limitations of the fiber material, since the light is confined and propagates in a hollow core region, i.e., in air. The ability of the HCPF to act as “substrate” and host active and noble gases, has enabled new research directions within the nonlinear fiber-optics field^13,14^. By changing the type of gas and its pressure, both the fiber dispersion and nonlinearity can be tuned^13,14^. Hollow-Core Anti-Resonant Fibers (HC-ARFs) are a sub-category of HCPF defined by broadband transmission and relatively low-loss^15^. These properties, combined with the high laser damage threshold due to a very small overlap of the light with the solid glass material, makes gas-filled HC-ARFs perfect candidates towards ultrafast applications, such as pulse compression^16^, multi-octave spanning SC generation^17–19^, and tunable DUV sources through resonant DW emission^20^. It has, for example, been shown to efficiently generate high-energy few femtosecond (fs) DW pulses in the DUV and vacuum UV^17,21^, which would have a number of important applications^13,14,22^. It should be noted that high energy DW pulses have been also reported using simple gas-filled capillaries instead of HC-ARFs, but due to their large core size, they require much higher pump pulse energy and peak power than in HC-ARFs^23,24^.

A key issue in almost any application of lasers and SC sources is their noise properties. Standard SC sources commercially available use long pump pulses (picosecond or nanosecond) to achieve high average power and have consequently been demonstrated to have high RIN both when pumped in the anomalous dispersion region just above the ZDW (modulational instability or MI based)^25^ and in the normal dispersion region just below the ZDW (Raman scattering based)^26^. In other words, MI and Raman scattering are equally noisy processes. The noise of the conventional MI based SC sources is further strongly increased by the subsequent generation of hundreds of solitons that interact in a highly phase and amplitude dependent way. This means two things: First of all the original noise seeding the MI, whether it is quantum or laser technical noise, becomes to some extent irrelevant due to the strong contribution from soliton collisions. Secondly the noise can, to a certain extent, be reduced by special fiber under-tapering to clamp the solitons and make them spectrally aligned^27^. Another standard way to strongly reduce the effect of SC noise in applications, such as imaging and spectroscopy, is to use high repetition rates to average out the noise^28^.

**Figure 1:**
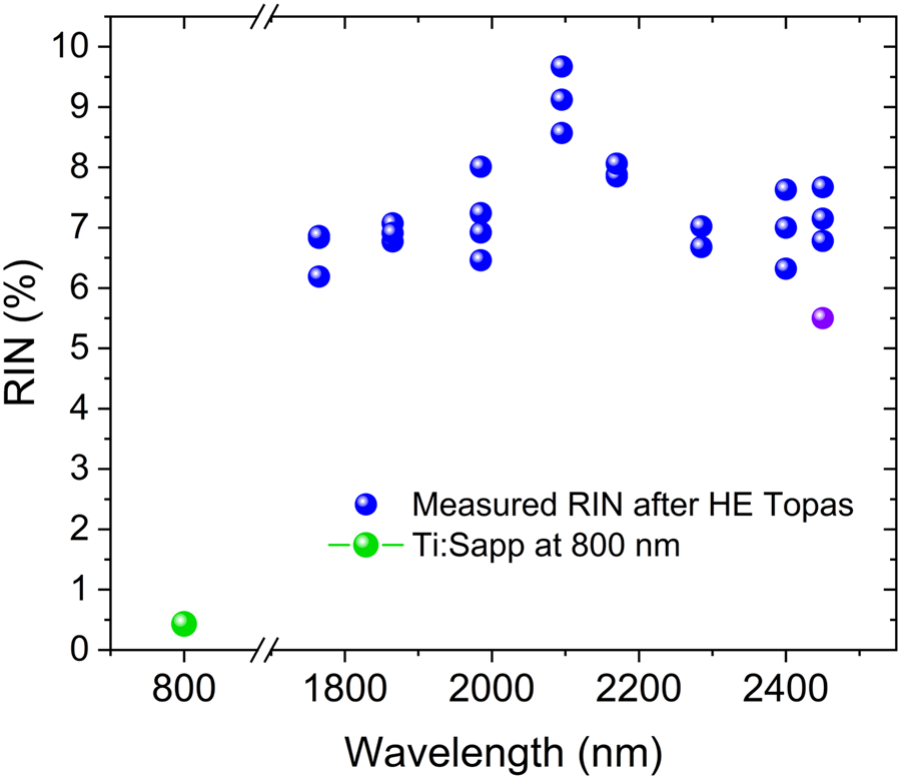
Experimental measurement of RIN for the laser system used in our experiments: Blue dots show the RIN at various OPA tuned wavelengths. Purple dot shows the value measured during SC generation and for numerical simulations. Green dot shows the RIN of the 800 nm Ti:Sapphire laser.

High repetition rates is generally not an option in gas-filled HCPF-based UV SC sources, because high peak power is required to achieve the gas-ionization necessary for SC generation. Except an interesting recent report where MHz-pumped UV SC generation in HC-ARFs demonstrated by compressing an ytterbium fiber laser from 300 fs to 25 fs^29^, most of the reports in gas-filled HCPF-based UV SC and DW generation have been using bulky fs lasers with kHz repetition rates. This means that the noise is of fundamental and crucial importance to ascertain the relevance of this new technology for applications. Whereas Ti:sapphire mode-locked lasers at 800 nm are very stable (RIN < 0.5%), the noise at longer wavelengths of tunable Optical Parametric Amplifier (OPA) based fs laser systems pumped by Ti:sapphire lasers is generally higher due to the series of nonlinear processes and stochastic gain variations involved in the generation of light at longer wavelengths^30^. In Fig. 1 we demonstrate this by showing the measured RIN of a standard tunable fs laser system using a HE-Topas module pumped by a Ti:Sapphire laser (Spitfire®Ace). The 800 nm pump laser has a noise of only 0.43%, but the output beam at >1700 nm has a RIN around 5.5–10% (see method section for experimental details).

Unfortunately there has been no experimental report yet on the pulse-to-pulse noise and spectral stability of these sources. Relying upon strong initial self-phase modulation (SPM), which is known to be a coherent effect, several papers have claimed high stability of the SC^13,14,22,31^, which has been supported by numerical modelling showing perfect SC coherence^17,32,33^. However, these numerical investigations have neither considered polarization effects nor the noise of the pump laser.

In conventional SPM-based fs-pumped SC generation in solid-core fibers with all-normal dispersion (ANDi), the impact of polarization mode instability (PMI) was demonstrated to be strong in non-polarization maintaining (non-PM) fibers, significantly reducing the pulse and fiber length below which good coherence can be obtained^34^. In most studies of gas-filled HCPF-based UV SC generation, the pump pulses have been shorter than 50 fs, so the PM properties might not play a big role. However, the pump laser noise is critical in any case and its effect on SPM-based fs-pumped SC generation far exceeds the effect of standard quantum noise^35^ hitherto used in all numerical noise studies of gas-filled HCPF-based UV SC generation. Furthermore, the fact that the wavelength of the DUV DW generated in gas-filled HCPF-based SC generation is determined by a power-dependent phase-matching condition, implies that any fluctuations of the pump power could directly translate into fluctuations of the wavelength and power of the DUV DW, provided of course that the nonlinear power-dependent term is sufficiently strong. It was for example already demonstrated that the power of the pump could be used to tune the DUV DW^20^, which strongly underlines the importance of a more thorough study of the SC noise, which takes into account the pump laser noise.

Here, we therefore present an experimental and numerical study of the RIN and the long-term stability of gas-filled HC-ARF-based UV SC sources pumped in the mid-IR with a relatively noisy pump laser. In particular we focus on the RIN of the generated DUV DW and its stability over a duration of 110 hours. We measured the RIN of the DW at 275 nm and compared it with numerical simulations, taking into account both the quantum noise and the actual pump laser fluctuations. It is important to note that although our Ti:sapphire laser at 800 nm has a measured RIN of only 0.43%, the RIN after the OPA was measured to have a relatively high noise of about 5.5% at 2.45 µm (see Fig. 1). Thus, the absolute values of the RIN we measure with this mid-IR pump laser are not representative for Ti:Sapphire pumped gas-filled HC-ARF-based UV SC sources, which will be significantly lower. However, the general message should apply for any pump laser, i.e., that the noise of such a type of UV SC source, with a UV part directly determined by a phase-matching condition, can be strongly influenced by the noise of the pump laser. The simulated results are in good agreement with the measured RIN, clearly underlining the importance of pump laser fluctuations and that these sources are not as coherent as is often believed. Furthermore, we support our results by an analytical discussion of the influence of the laser fluctuations on the phase matching conditions.

## Experimental part

**Figure 2:**
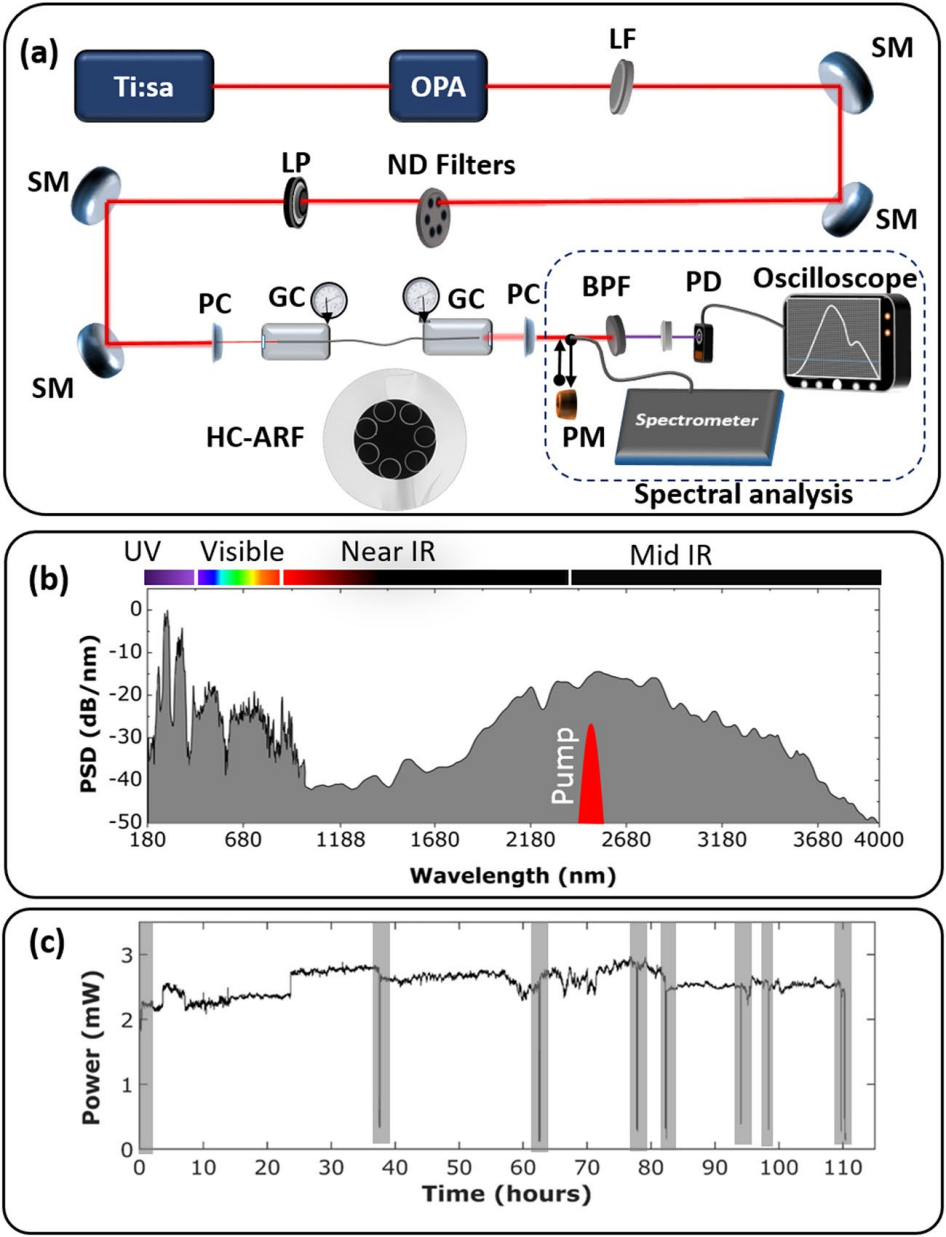
(**a**) Experimental setup with a Ti:Sapphire laser pumping an OPA, long pass filter (LF), silver coated mirror (SM), neutral density filters (ND), linear polarizer (LP), CaFl_2_ Plano-convex lenses (PC), gas cells (GC), power meter (PM), bandpass filter (BPF), photodiode (PD). Bottom inset: Scanning Electron Microscopy image of the HC-ARF with 44 µm core diameter. (**b**) Power Spectral Density (PSD) of the generated SC. (**c**) Total output power versus time over 110 hours. Every dip, highlighted with shaded gray, indicates the time of a spectral measurement (change of the beam path from power meter to fiber probe of the spectrum analyzer).

A single-ring HC-ARF with a 44 µm core diameter and 7 non-touching capillaries (Fig. 2) is filled with argon at 27 bar and pumped in the anomalous dispersion regime at 2.45 μm with ~100 fs (T_FWHM_) and ~8 μJ pulse energy at 1 kHz repetition rate. The set-up used in our experiments is shown in Fig. 2(a). The dispersion and loss profile of the HC-ARF used in our experiments can be found in^17^, from which it is seen that the pump wavelength is in the anomalous dispersion region inside a low-loss transmission window. An SC spanning from 180 nm to 4 µm is generated, as seen in Fig. 2(b), which has a strong DUV DW at 275 nm followed by a weaker spectral peak at 360 nm.

**Figure 3:**
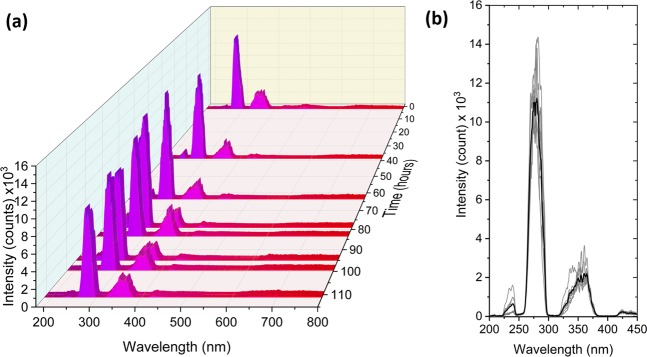
(**a**) Long-term stability of the DUV part of the SC spectrum measured over 110 hours. (**b**) Overlay of the measured spectra in grey. The black spectrum signifies the mean of the 8 recorded spectra.

The average output power of the SC was monitored with a thermal power meter (Thorlabs, C-series) for 110 hours. Minor power fluctuations were observed during the measurement, but without any significant decay, as seen in Fig. 2(c). The DUV spectral profile was recorded at 8 instances over the 110 hours, corresponding to the 8 dips in power marked by a gray shaded region in Fig. 2(c). The spectra are shown separately in Fig. 3(a) and overlaid as gray lines in Fig. 3(b), with the mean spectrum marked by the black curve, which clearly indicates the spectral power fluctuations. These fluctuations are at the heart of this work and will be characterized in the following in terms of the RIN.

The RIN was statistically computed by tracking the peak of every recorded pulse (corresponding to maximum voltage) in the oscilloscope after the noise floor level (reference) has been subtracted (see method section for more details). The RIN = σ/μ is then defined as the standard deviation ($\sigma$) of the amplitude of the peaks divided by the mean (${\rm{\mu }}$) of the amplitude of the peaks.

**Figure 4:**
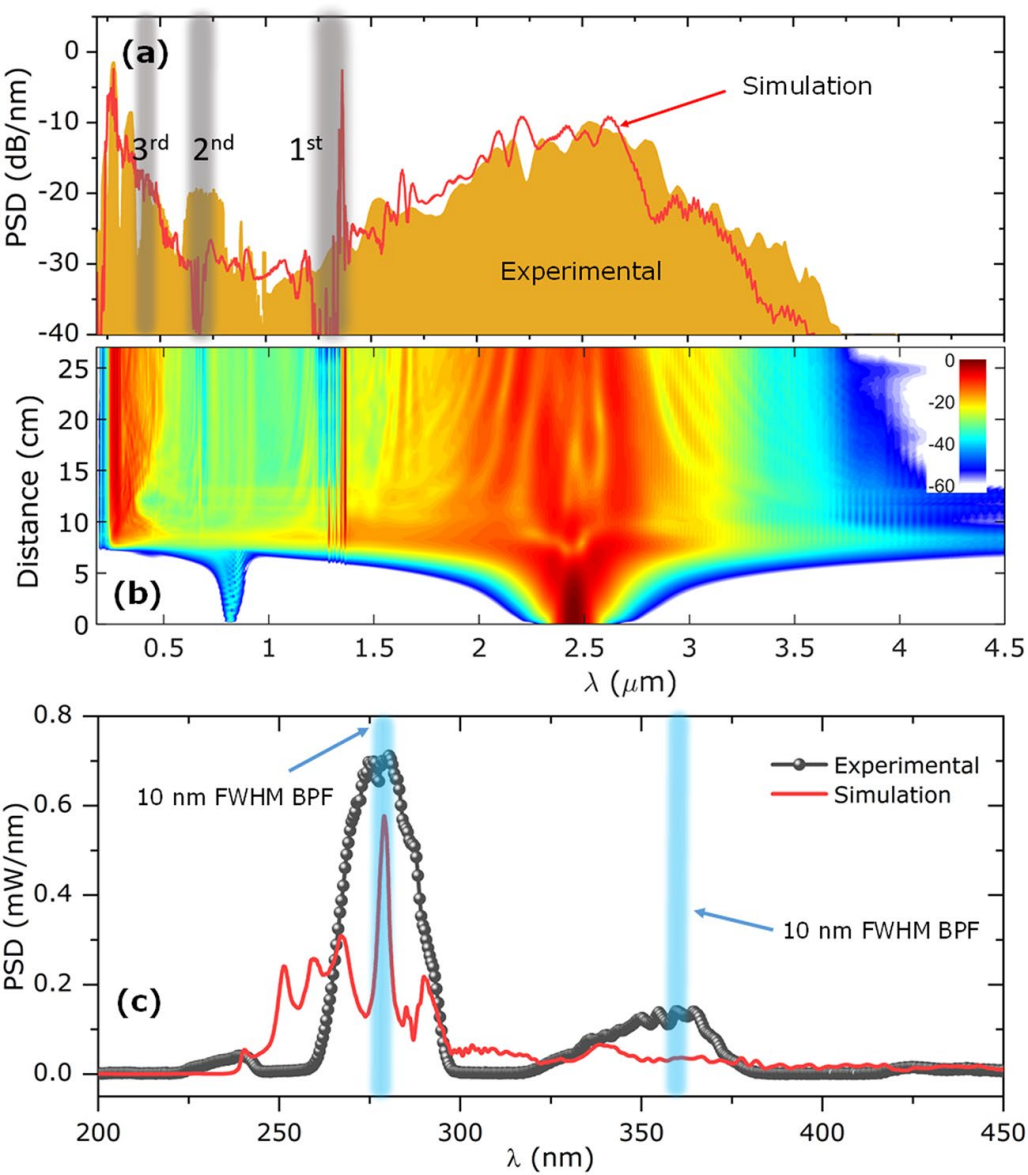
(**a**) Numerical simulation and experimental spectrum of broad SC generated in HC-ARF, with gray shade showing the first 3 resonances of the HC-ARF. (**b**) Evolution of spectrum along the length of fiber. (**c**) Numerical and experimental spectra, plotted in a linear scale. Two filters with 10 nm FWHM (shown in blue) are used to measure RIN at 280 nm and 360 nm.

The RIN of the DUV DW at 275 nm was in this way measured to be 33.3%. The RIN of the peak at 360 nm was measured using the same procedure described in the method section, with a 10 nm bandwidth filter centered at 360 nm and a fast silicon photodiode (NewFocus, Model 1801, 125 MHz bandwidth). 10,000 pulses were recorded, similar to the measurements performed at 280 nm (filtered spectra in supplementary Fig. S2). The RIN at 360 nm was found to be 8.84%. The increase in noise from the internal peak at 360 nm to the 280 nm peak at the spectral edge follows the typical trend of SC sources pumped in the anomalous dispersion region, i.e., that the noise of the SC increases towards the edges of the spectrum due to fundamental nonlinear soliton-DW effects [25]. However, another possible purely experimental explanation as to why the RIN of the 280 nm peak is higher is that the spectrum in that region is not as flat as that at 360 nm. When a section of the spectrum is filtered (with a 10 nm FWHM filter for example), the RIN will tend to be lower if the spectrum is flat. From Fig. 4(c) it is seen how a slight shift of the narrow 280 nm peak will tend to move the peak out of the filter region, whereas slight changes to the broad 360 nm peak will not have a noticeable effect on how much light is in the filter bandwidth.

The experimentally observed pulse-to-pulse RIN, measured here for the first time for this type of DUV SC source, contradicts the theoretical predictions and numerical conclusions of earlier papers claiming perfect stability^13,14,22^ and others presenting numerical modelling of the coherence, showing a perfect coherence of 1^17,20,32,33^. Some de-coherence across the SC was observed in a recent paper, which we will discuss in the last section of the article, but the conclusion was that the UV DW remained largely coherent^20^.

Of course one factor is the relatively high noise of 5.5% of our pump laser, but even if it was reduced by a factor of ten to the Ti:Sapphire level, this would not bring the noise level to zero as for perfect coherence. The key factor explaining this contradiction is that the earlier modelling of such UV sources did not take into account laser technical noise, i.e., the RIN of the pump laser. Recent numerical and experimental work on SPM-based SC generation with fs pulses in solid-core ANDi fibers^35,36^, has however clearly demonstrated that laser technical noise of just 0.5–1% is strongly dominating quantum noise. Physically it therefore makes sense that the noise of the DW is significant.

## Theory and experiments of RIN

To understand the physics behind this noise performance and validate its strength, we simulate the SC generation taking both quantum noise and our measured laser technical noise into account in the initial condition. We use the standard unidirectional pulse propagation equation, which accounts for the plasma effect^33,37,38^:

1 $$\frac{\partial {E}}{\partial {z}}={i}\left[{\beta }({\omega })-\frac{{\omega }}{{{v}}_{{g}}}+{i}\frac{{\alpha }({\omega })}{2}\right]{E}+{i}\left[\frac{{{\omega }}^{2}}{2{{c}}^{2}{{\varepsilon }}_{0}{\beta }({\omega })}\right]\hat{{F}}\{{{P}}_{{NL}}\}$$

where *z* is the propagation distance along the fiber, *t* is the time in a reference frame moving with the pump group velocity *v*_*g*_, *E* = *E*(*z*,*ω*) is the electric field in the frequency domain defined over the effective mode area^39^(here assumed to be constant because it only varies a few percent over the wavelength span), *ω* is the angular frequency, *α(ω)* is the linear propagation loss of the fiber, *c* is the speed of light in vacuum, β *(ω)* is the propagation constant, and $\hat{F}\{{P}_{{NL}}\}$ represents the Fourier transform of the nonlinear polarization *P*_*NL*_*(z,t)* = *ε*_0_*χ*^(3)^*E(z,t)*^3^ + *P*_*ion*_*(z,t)*^37,38^. The first term is the Kerr effect, where *ε*_0_ is the vacuum permittivity, and *χ*^(3)^ is the third-order nonlinear susceptibility of the noble gas, which here is argon. The second term describes the nonlinear polarization due to molecular or atomic ionization^18,37,38,40^, in which the free electron density was calculated using the quasi-static tunneling ionization approximation and the Ammosov, Delone, and Krainov (ADK) model, described in^41^. Full details of the model and arguments for the used approximations may be found in our earlier paper^17^.

A reasonable prediction of the wavelength of the UV DW can be found by estimating the phase-mismatch Δβ = β_*DW*_ − β_*sol*_ between the propagation constant of the DW (β_*DW*_) and the soliton (β_*sol*_). It should be noted that any relation made to Nonlinear Schrödinger (NLS) solitons and the typical nonlinear parameter *γ* is referring to the underlying Generalized Nonlinear Schrödinger (GNLS) type envelope model that does not involve ionization^22^. From the GNLS model the phase-mismatch between the soliton at the pump frequency *ω*_0_ and the DUV DW at the frequency *ω* is given by^42,43^:

2 $$\Delta {\beta }({\omega })\approx {\beta }({\omega })-{{\beta }}_{0}-({\omega }-{{\omega }}_{0}){{\beta }}_{1}-{{\beta }}_{{sol}}$$

where β_0_ is the propagation constant and β_1_ = *d*β*/dω = 1/v*_*g*_ is the inverse group velocity, both evaluated at the pump frequency *ω*_0_. Several versions of this phase-matching condition for the UV DW generated in gas-filled HCPFs have been proposed using different approximations^20,38^. Here we use the exact *N*-soliton solution to the underlying integrable NLS equation in the GNLS model (obtained by considering only second order dispersion β _2_ and the Kerr effect), which is a bound state between *N* fundamental solitons with different propagation constants. We match to the one with the largest propagation constant β_sol_ ≈ β_sN_ = (2N − 1)^2^/(2L_D_)^44^ where L_D_ = T_0_^2^/ | β_2_ | is the dispersion length and *T*_0_ is related to the FWHM as *T*_*FWHM*_ = *T*_0_ *ln(1* + *√2)*. This means that we have neglected the effect from the ionization, which is a good approximation for the UV DW^38^. In the supplementary material we compare all the different versions of the phase-mismatch in terms of validity and their predictions of the UV DW wavelength, showing that Eq. (2) provides a slightly better match to the experiment. The phase-mismatch (plotted in the supplementary Fig. S1) predicts a DW wavelength of 238 nm, which is in relatively good agreement with the experimentally measured DW at 275 nm, given that the 2450 nm pump wavelength is far away from the ~1600 nm ZDW and given the many approximations used (see supplementary Fig. S1).

In our calculation, we included both quantum noise and the measured 5.5% pulse-to-pulse amplitude and pulse width fluctuations from the laser as in^35^. The initial condition with the noise terms becomes:

3 $${E}(0,{t})=\sqrt{{{P}}_{0}(1+{\Delta }_{{P}})}\exp \left[\frac{-{{t}}^{2}}{2{[{{T}}_{0}(1+{\Delta }_{{T}})]}^{2}}\right]+{\hat{{F}}}^{-1}\{{\Delta }_{{Q}}\}$$

Here *T*_0_ is the pulse duration (60 fs = T_FWHM_/$\sqrt{4{ln}2}$), *P*_0_ is the peak power (estimated to be 75 MW), and ${\hat{F}}^{-1}$ is the inverse Fourier transform. The quantum noise *Δ*_*Q*_ of Eq. (3) is modeled semi-classically as the standard one-photon-per-mode (OPPM) noise added to the initial condition in the Fourier domain as one photon of energy ℏω_m_ and random phase *Φ*_*m*_ in each spectral bin m with angular frequency *ω*_*m*_ and bin size *ΔΩ*^45^. The OPPM noise in the frequency domain is given by $\triangle Q=\sqrt{h{\omega }_{m}/\Delta \Omega }$ exp(i2πΦ_m_), where *h* is Planck’s constant and *Φ*_*m*_ is a random number uniformly distributed in the interval [0,1]. The RIN Δ_*P*_ is Gaussian distributed white noise with zero mean and standard deviation 5.5%. To take into account that our Ti:Sapphire pump laser is a mode-locked laser, we assume that the peak power and pulse length are anti-correlated, i.e., Δ_T_ = −ηΔ_P_ , where η = 1.0 is chosen. Recently a Onefive Origami 10 fs laser was studied, for which η = 0.8, and anti-correlated amplitude and pulse length noise of only 0.2% was shown to strongly dominate quantum noise^35^. In ref. ^36^, η = 1.0 and an experimentally measured pump RIN of 1% was used and shown to correctly give the measured noise around the pump.

We numerically calculated the spectral profile of both the RIN and the coherence using 100 spectra from 100 runs with different seeds in the ensemble. When laser technical noise is ignored, the coherence is perfect (see supplementary material Fig. S4), but when the 5.5% RIN is taken into account the coherence is destroyed and the RIN is high, as anticipated (see supplementary material Fig. S5). A direct comparison of the 100 SC spectra overlaid each other clearly shows the significant difference for the two cases (see supplementary material Fig. S6).

In Fig. 4(a,c) the experimental and numerical average spectra (with both noise sources taken into account) are compared and we see that the numerical model accurately captures the spectral bandwidth and the DW at 275 nm, but not the internal peak at 360 nm. Figure 4(b) shows the standard spectral evolution along the fiber, dominated by SPM of the pump and generation of the UV DW once the maximum compression point is reached at ~7.5 cm fiber length.

**Figure 5:**
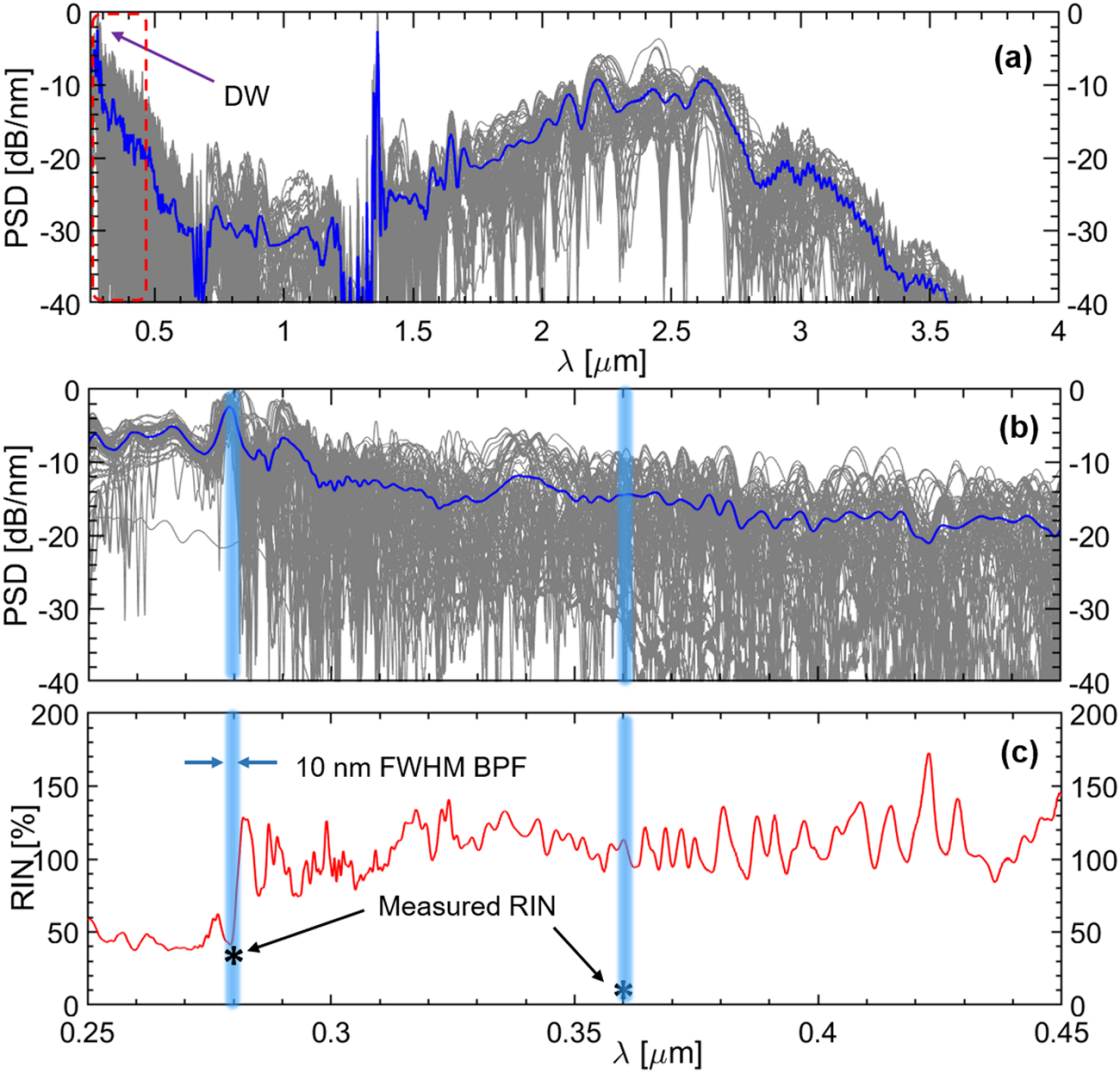
Numerical simulations: (**a**) the SC generated by pumping an HC-ARF with ~100 fs pulses at 2450 nm and 27 bar Ar pressure. 100 realizations were computed. Blue spectrum shows the average of the realizations. (**b**) A zoom of the UV section of the spectra. Experimentally the RIN was measured in the blue regions at the DW at 280 nm and at 360 nm. (**c**) Calculated RIN for the 100 realizations plotted in red, with stars indicating the measured 33.3% and 8.84% at 280 nm and 360 nm, respectively. The numerically calculated RIN at 280 nm was found to be 35%.

In Fig. 5(a,b) we show the numerically calculated average and individual SC spectra with both noise sources taken into account, including a zoom of the UV spectral region. From the pulse-to-pulse statistics, we calculate the RIN shown in Fig. 5(c). In particular we obtain 35% RIN at 280 nm, which matches very well the experimentally found 33.3%. Since negligible noise was found when using only quantum noise (see supplementary Fig. S4), this strongly suggests that laser technical noise is the main reason of the final poor noise performance. Since the internal part of the spectrum at around 360 nm did not perfectly match with our numerical simulations, which is often the case^20,32^, the numerically and measured RIN at 360 nm cannot be directly compared.

## Discussion and Conclusions

In this work we presented the first experimental study of the long-term stability and pulse-to-pulse noise properties of a DUV SC source based on noble gas-filled HC-ARFs. We found that the spectrum and total power fluctuated, but did not show sign of decay over 110 hours of operation. However, our experiments showed that the pulse-to-pulse RIN of the DUV DW at 275 nm wavelength was 33.3%, which is much higher than predicted or found by numerical modelling in earlier reports on gas-filled DUV SC generation^13,14,17,20,22,31–33^.

We have argued from numerical modelling that the observed strong noise originates from the RIN of the pump laser, which was measured to be 5.5%. Our modelling with only standard weak quantum noise (see supplementary fig. S4) confirmed near to perfect coherence (i.e., negligible noise) at all wavelengths, just as in the earlier reports where only this type of noise is considered^14,17,20,32,33^. In contrast we found a RIN of 35% of the DUV DW when taking into account laser technical noise, which is in good agreement with the experiments. This general result means that the laser technical noise is ultimately limiting the noise performance of SC sources based on coherent fs-pumped SPM, which is in line with recent demonstrations of the noise of SC sources using fs pulses to pump solid-core fibers with all-normal dispersion^34–36^. Earlier studies have also reported a strong amplification of pump laser noise in SC generation in solid-core microstructured fibers by pumping in the anomalous regime with femtosecond pulses^46,47^. However, in these cases there is a strong contribution from the Raman effect of silica on the SC generation, which is known to introduce noise, and thus it is less surprising that the SC spectrum is noisy. In our case the SC generation takes place in a Raman-inactive noble gas.

It was found in a recent publication, using numerical modelling with only quantum noise, that the coherence could be not perfect in these DUV SC sources^20^. It is important and very interesting to put the results of this paper into context with our results in terms of soliton numbers and known properties of SC generation. The first key point is that fs-pumped soliton fission based SC generation can be just as noisy as long-pulse pumped MI based SC generation for large soliton numbers *N*, specifically when *N* > 16^48^. The second key point is that in MI-based SC generation any weak noise seed is enough to trigger MI and generate the typically high number of solitons, whose subsequent random interaction will dominate the SC noise. The type and particular strength of the weak seed noise is not important.

In the modelling in^20^ 38 fs Gaussian shaped pulses from an λ_0_ = 800 nm (Ti:Sapphire) laser was used to pump an HCPF with a core diameter of D = 44 μm filled with argon at a pressure of 13.5 bar. From Fig. 26 in^14^ this gives a nonlinear refractive index of n_2_ = 1.2 × 10^−22^ m^2^W^−1^. Assuming that the effective area A_eff_ is the core area, the nonlinear coefficient is then γ = ω_0_n_2_/(cA_eff_) = 8n_2_/(λ_0_D^2^) = 1.65 × 10^−6^ (Wm)^−1^. From Fig. 1 in^20^ we find a group velocity dispersion of β_2_ = −2 fs^2^/cm, which gives a soliton number of *N* = 17.9 and 12.6 for the two pulse energies of 3.0 μJ and 1.5 μJ used in their modelling, respectively. This means that the high pulse energy case in which de-coherence was observed in^20^ has a soliton number that was in fact above 16, which is known to lead to highly noisy SC generation for even the very weak quantum noise. The low pulse energy case had a soliton number below the 16 and should thus be highly coherent if only weak quantum noise is considered.

Based on our experimental parameters, which are ~100 fs pulses, argon at 27 bar, D = 44 μm, and λ_0_ = 2450 nm, given the n_2_ = 2.069 × 10^−22^ m^2^W^−1^ from^14^ and β_2_ = −82.5 fs^2^/cm from^17^, we find a soliton number of *N* = 4.66. Thus we are in the low soliton number case, where complete SC coherence would be expected when using fs-pumped soliton fission based SC generation and taking only the very weak quantum noise into account. Our experiments, confirmed by numerical modelling, demonstrates how pump laser fluctuations, against this expectation, makes the DUV DW and SC have high noise.

Our results clearly reveal the importance of using a low-noise pump laser for DUV SC sources based on gas-filled HC-ARF. Our pump at 2450 nm has a RIN of 5.5% due to how it is generated from the 800 nm Ti:Sapphire seed. However, the mode-locked Ti:Sapphire laser itself, which is the laser most often used to pump these DUV SC sources, has a much lower RIN (in our case it was measured to be 0.43% - see supplementary material S3) and would thus be much more suitable as pump laser. However, a pump laser RIN of 0.43% is still a much stronger noise source than quantum noise for SPM-based SC generation^33^, and thus even the Ti:Sapphire pump laser would have to be stabilized to truly enable a future coherent DUV SC source.

## Methods

The gas filling method is similar to that presented in our previous work^17^, where two ends of the HC-ARF are cleaved with a tension cleaver and then sealed into a custom-made high pressure gas cells. The pressure is kept constant at 27 bars throughout the stability measurement and noise characterization.

The output power is monitored with a thermal power meter and the value is logged in a computer for the entire duration of 110 hrs.

For the RIN measurement; The generated SC from the HC-ARF is collimated and filtered with a 10 nm FWHM bandpass filter with center wavelength at 280 nm (filtered spectra are shown in the supplementary Fig. S2). A narrow bandwidth filter is chosen because SC noise is averaged out with respect to the bandwidth of the filter, i.e., a large bandwidth tends to give lower noise^4^. The filtered SC is then sent to a fast Si detector (Thorlabs DET102, 350 MHz bandwidth, 1 ns rise time) and it was ensured that all power levels were within the linear operation regime of the detector. To determine the RIN, a train of 10,000 pulses (or voltage-time series) was recorded with a fast oscilloscope (Teledyne LeCroy - HDO9404 −10 bits resolution, 40 Gs/s, and 4 GHz bandwidth). For each pulse, the noise floor is calculated and subtracted from the peak. It is important to note that this setup enables the measurement of pulse-to-pulse intensity fluctuations since the photodiode and oscilloscope are fast enough to detect individual pulses of the 1 kHz SC^49^.

## Supplementary information

Supplementary information.
