# Supplementary material for: Noise and spectral stability of deep-UV gas-filled fiber-based supercontinuum sources driven by ultrafast mid-IR pulses
Source: Sci Rep. 2020 Mar 18;10:4912. doi: 10.1038/s41598-020-61847-w (PMC7080841; doi:10.1038/s41598-020-61847-w)
Supplement: Supplementary file 1 — Supplementary information. [file 41598_2020_61847_MOESM1_ESM.pdf]

Supplementary material

Noise and spectral stability of deep-UV gas-filled fiber-based supercontinuum sources driven by ultrafast mid-IR pulses

Abubakar I. Adamu, [1, a)] Md. Selim Habib, [2] Callum R. Smith,[1] J. Enrique Antonio Lopez, [2] Peter Uhd Jepsen, [1] Rodrigo Amezcua-Correa, [2] Ole Bang, [1, 3, 4] and Christos Markos[1, 4, b)]

[1]DTU Fotonik, Technical University of Denmark, Kgs. Lyngby, DK-2800, Denmark
[2]CREOL, The College of Optics and Photonics, University of Central Florida, Orlando, FL-32816, USA
[3]NKT Photonics, Blokken 84, Birkerød 3460, Denmark
[4]NORBLIS IVS, Virumgade 35D, DK-2830 Virum, Denmark.
[a)] Electronic mail: abisa@fotonik.dtu.dk
[b)] Electronic mail: chmar@fotonik.dtu.dk

This document provides supplementary information to "Noise and spectral stability of deep-UV gas-filled fiber-based supercontinuum sources driven by ultrafast mid-IR pulses". Here we provide the details of the phase-matching conditions between the soliton and dispersive waves, and compare our expression to other expressions in the literature. Additionally, we provide the figures for filtered DUV used for the RIN measurements as well as the histograms of the RINs for the pump laser, the Ti:sapphire laser, and the DUV spectrum at 360 nm and 280 nm. We further compared the coherence and RINs when the pump laser noise is not considered and when considered.

I. PHASE-MATCHING CONDITION BETWEEN SOLITON AND DW

In the numerical studies of UV SC and DW generation in gas-filled HCPFs the most used model is the unidirectional pulse propagation equation (1) for the full electric field, defined over the effective mode area A_{eff}, which includes the higher harmonics. The model (1) accounts for the full dispersion, the Kerr nonlinearity, and the plasma effect. However, in most theoretical considerations of, e.g., soliton and DW interaction, and also in some general modelling [1], the more standard GNLS envelope equation is used, which in the time domain is given by [2]

$$i\frac{\partial A}{\partial z}+ i\frac{\alpha}{2}A-\frac{\beta_2}{2}\frac{\partial^2 A}{\partial t^2}-i\frac{\beta_3}{6}\frac{\partial^3 A}{\partial t^3}+\gamma\left(1+is\frac{\partial}{\partial t}\right)|A|^2A = 0 \qquad (\boldsymbol{S1})$$

Here self-steepening is present through the parameter $s=1/\omega_0$, where ω_0 is the pump frequency, the Kerr effect is present through the nonlinear parameter $\gamma=n_2\omega_0/(cA_{eff})$, where n_2 is the material nonlinearity, and linear fiber loss is present through the parameter α, but the Raman effect is absent, because only noble gasses are considered. Second- and third order dispersion have been included through the parameters $\beta_n= d^n\beta/d\omega^n|_{\omega=\omega_0}$, where $\beta(\omega)$ is the linear propagation constant of the fundamental guided mode. The application of two different models has lead to more than one version of the important phase-matching condition for soliton-DW interaction, which we would like to discuss here. The phase-mismatch $\Delta\beta$ is generally defined as the difference between the propagation constant β_{DW} of the DW at frequency ω and the nonlinear propagation constant β_{NL} of the soliton at frequency ω_s. Assuming that the soliton is the pump, $\omega_s=\omega_0$, the phase-mismatch becomes

$$\Delta\beta(\omega)=\beta_{DW}(\omega)-\beta_{NL}(\omega_0)=0 \qquad (\boldsymbol{S2})$$

In both models people agree on that the DW propagation constant is given by

$$\beta_{DW}(\omega)=\beta(\omega)-\beta_0-(\omega-\omega_0)\beta_1 \qquad (\boldsymbol{S3})$$

where $\beta_0 = \beta(\omega_0)$ and $\beta_1= d\beta/d\omega|_{\omega=\omega_0} = 1/v_g(\omega_0)$ is the inverse group velocity, both evaluated at the soliton frequency ω_0. At present, in the literature there exists two different versions of β_{NL}. If one wants

to study DW generation as resonant energy transfer from a soliton, then one needs a soliton solution, which is generally found as the fundamental NLS soliton solution to Eq. (S1) with $\alpha=\beta_3=s=0$, i.e., ignoring loss and all higher order dispersion and nonlinear effects. The NLS soliton solution has peak power P_0, pulse length T_0, and propagation constant $\beta_s=\gamma P_0/2$, and is given by

$$A(z,t)=\sqrt{P_0}\,\mathrm{sech}(t/T_0)\exp(i\beta_{s1}z) \qquad \textbf{(S4)}$$

Thus, considering phase-matching to the fundamental NLS soliton, one would get $\beta_{NL}=\beta_{s1}=1/(2L_D)$ and since the dispersion length $L_D=T_0^2/|\beta_2|$ is equal to the nonlinear length $L_{NL}=1/(\gamma P_0)$ for the fundamental soliton then $\beta_{NL}=\gamma P_0/2$ [3,4]. It is important that this expression is only valid for a fundamental soliton with soliton number N=1, disregarding that the pump pulse typically is stronger and has a higher soliton number N>1, where the soliton number is defined by the relation $N^2=L_D/L_{NL}$. Travers *et al.* realized that this definition of β_{NL} would give too weak a nonlinear contribution to the mismatch and replaced it with P_C, defined as the peak power at the point of maximum compression [5,6], which was then estimated to be $P_C=4.6NP_0$ from numerical modelling based on Eq. (S1)[6]. This gives the following expression for the phase-mismatch

$$\Delta\beta(\omega)=\beta(\omega)-\beta_0-(\omega-\omega_0)\beta_1-\frac{\gamma P_C}{2}=0,$$

$$P_C\ =4.6NP_0 \qquad \textbf{(S5)}$$

We note that P_C actually has been estimated analytically to be $P_C=NP_0\sqrt{2}$, which has been shown to accurately represent the maximum power of a higher-order soliton for large soliton orders, i.e., the peak power at the point of maximum compression [7]. We have verified this with the N=8 soliton and found $P_C=NP_0\sqrt{2}$ to be very accurate, whereas $P_C=4.6NP_0$ provides a too high estimate. However, we take a more accurate approach and consider the exact analytical N-soliton solution to the NLS equation [4], which is a bound state of N fundamental 1-solitons with propagation constants $\beta_{sn}=(2n-1)^2\beta_{s1}$, where n=1,2,…,N. The n=N soliton with the largest propagation constant $\beta_{sN}=(2N-1)^2\beta_{s1}$ is also the one with the largest amplitude and smallest pulse length[4], which means that it has the broadest spectrum and is thus the one that spectrally overlaps the most with the DW. We therefore naturally use this propagation constant as β_{NL}, which gives our phase-mismatch

$$\Delta\beta(\omega)=\beta(\omega)-\beta_0-(\omega-\omega_0)\beta_1-\frac{(2N-1)^2|\beta_2|}{2T_0^2}=0, \qquad \textbf{(S6)}$$

The definition of β_{NL} as the propagation constant of a soliton means that effects, such as the ionization and self-steepening cannot be taken into account, since no expression for the soliton solutions exists when these higher order terms are taken into account. Using instead a simple plane-wave ansatz to find the nonlinear propagation constant directly from the field equation (1) and then afterwards adjusting the Kerr term to include the nonlinear parameter γ Novoa *et al.* derived the following expression for the phase-mismatch [8]

$$\Delta\beta(\omega)=\beta(\omega)-\beta_0-(\omega-\omega_0)\beta_1-\gamma P_C\frac{\omega}{\omega_0}+\frac{\omega_0\rho}{2n_0c\rho_{cr}}\frac{\omega_0}{\omega}=0,$$

$$P_C\ =4.6NP_0 \qquad \textbf{(S7)}$$

where c is the speed of light in vacuum, n_0 is the linear refractive index of the gas at the pump wavelength, ρ is the free-electron density, ρ_{cr} is the critical free-electron density at which the plasma is opaque. The Kerr term takes into account shock formation and self-steepening through the factor ω/ω_0 and does not have the factor ½ since it is not found as a soliton solution, but through a plane-wave ansatz. The last term is the ionization term, which also takes into account the factor ω/ω_0 . Novoa et al. showed that the ionization term competes with the Kerr term and allows to explain the generation of mid-IR DWs, but is not very important for the UV DWs [8]. This is also seen in Fig. S1, in which we plot the different phase-mismatch curves (S5), (S6), and (S7). The full and dashed blue curves are thus very close at short wavelengths but not a longer wavelengths. It can also be seen from Fig. S1, that the plane-wave derived expression (S7) does not accurately predict the DW wavelength at which that the mismatch is zero, whereas Eqs. (S5) and (S6) both provide good predictions of the 275 nm DW wavelength observed experimentally, the expression (S6) being slightly better. In fact, due to the relatively weak contribution from the nonlinearity, the phase-mismatch curve when the nonlinear part is ignored (dashed black curve) looks very similar to the phase-mismatch curve we derived in Eq. (S6).

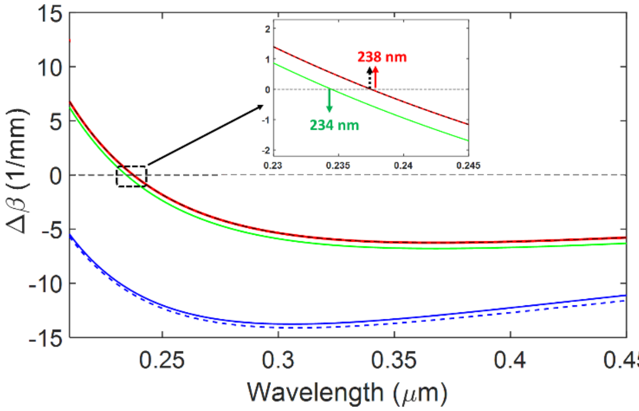

Fig. S1: Phase-mismatch curves as defined in Novoa et. al. [8] with both the Kerr and ionization effects (solid blue) and with only the Kerr effect (dashed blue). The solid green is the phase-mismatch curve described in Eq. (S5) as reported in[9]. The solid red curve is from the phase-matching condition (S6) used in this manuscript. The black dashed curve is (S6) when the nonlinear part is ignored.

The experimental parameters and laser specifications used to calculate the phase-mismatch and used in the dynamical modelling of the SC generation given below are: 100 fs pulse width (FWHM intensity), 2450 nm pump central wavelength and a pump pulse energy of 7.5×10^{-6} Joules. The effective area is $A_{eff}\approx1.65\alpha^2$, where α=22 µm is the core radius of the HC-ARF[9,10]. The dispersion is empirically calculated using [10] for Argon gas in a silica AR-HCF of 22 µm core radius, a tube diameter of 25 µm, with 7 non-touching tubes and a tube wall thickness of 640 nm. The pressure is 27 bar

and the temperature is 298 K. The free electron density is ρ = 3.87 x 10^{23} m^{-3} and the critical plasma density is ρ_{cr} = 1.8167 x 10^{26} m^{-3}. The nonlinear parameter is γ = 6.6447 x 10^{-7} $W^{-1}m^{-1}$.

II. SC generation and noise measurements

The experiment was carried out at ambient room temperature using a custom made gas-cells equipped with CaF_2 windows at one end for passage of incoming coupled light and another for output of SC light before collimation. Prior to taking measurements, the gas cells (interconnected with the hollow core fiber) are purged with 99.99% purity Argon gas to remove any impurity and ambient air from the chamber. To generate the multi-octave SC generation, the argon pressure was tuned to 27 bar by manually adjusting the compressed gas regulator and a fixed pressure is maintained throughout the stability measurements in Fig. 2(c) and Fig. 3.

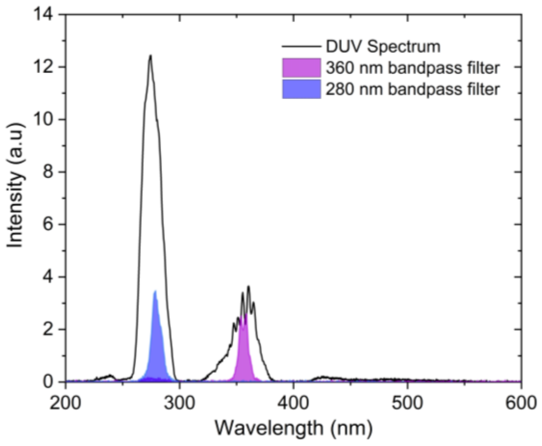

Fig. S2. The spectra of filtered DUV parts at 360 nm (purple) and 280 nm (blue), together with the full spectrum. Note: the intensity of the three spectra are not normalized. The FWHM of the filters is 10 nm.

After the broad SC is generated, we filter the 275 nm dispersive waves using a 280 nm central wavelength bandpass filter with >12% transmission in the bandpass window and a blocking wavelength range of 200 nm to 10,000 nm. Another filter with 360 nm central wavelength is used to filter the relatively low energy peak at 360 nm. The filtered light is shown in purple and blue in Fig. S2. The filtered light is then focused on a fast photodiode with 1 ns rise time, connected to an oscilloscope, where a Matlab script is used to acquire the peak of each individual pulse and its associated background noise level. A train of 10,000 pulses was captured for the RIN measurements, and the RIN was computed through the Matlab script. Histograms are shown in Fig. S3 with 3 different fits. For all measurements we do not use fits, but take the statistical values for the RIN, as some of the distributions are skewed and doesn't necessarily fit a Gaussian, normal, or gamma distribution.

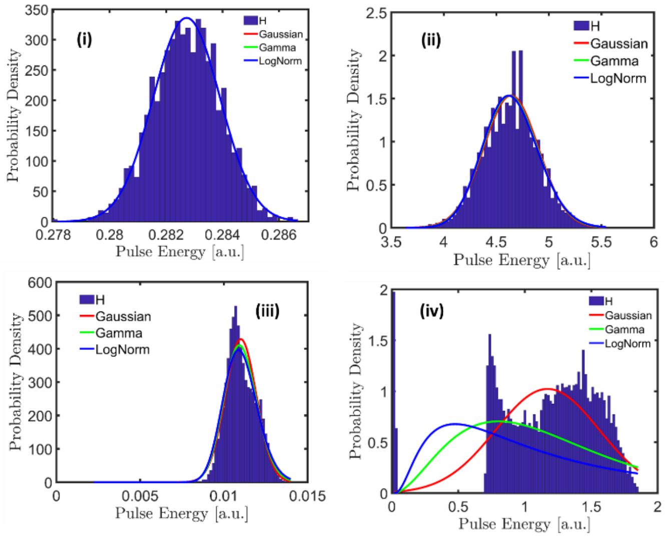

Fig. S3. Histograms of filtered pulse energy. (i) Ti:Sapphire laser at 800 nm with 0.43 % RIN. (ii) Pump at 2.45 µm after the TOPAS, with 5.5% RIN. (iii) 10 nm filtered 360 nm DUV SC part with 8.84 % RIN. (iv) 10 nm filtered 280 nm DUV SC part with 33.3 % measured RIN. Red, green and blue are gamma, Gaussian and lognormal fits. Note that the RINs used are the statistical values not fits.

III. CALCULATION OF SC COHERENCE AND RIN

The modulus of the complex degree of first order spectral coherence of the generated SC was calculated using the following expression[11]:

$$\left|g_{12}^{(1)}\right| = \left|\frac{\langle A_m^*(\omega)A_n(\omega)\rangle_{m\neq n}}{\sqrt{\langle|A_m(\omega)|^2\rangle\langle|A_n(\omega)|^2\rangle}}\right| \quad \textbf{(S8)}$$

The angle brackets in Eq. (S8) represent an ensemble average over the independent simulations m and n. The value of $\left|g_{12}^{(1)}\right|$ indicates the quality of the spectral coherence of the SC and is primarily a measure of the phase stability[12]. The spectral coherence would be perfect if $\left|g_{12}^{(1)}\right| = 1$, whereas $\left|g_{mn}^{(1)}\right| = 0$ indicates that the SC spectrum has a random phase fluctuation from shot to shot. The modulus of the complex degree of first order spectral coherence as a function of the propagation distance is shown in Fig. S4, for the case when only one-photon-per-mode (OPPM) noise is considered. It can be seen from Fig. S4 that the whole output spectrum is fully coherent in this case. However, when we add the pump laser noise of 5.5% (measured from the experiment), the spectral coherence of the generated SC drops drastically, which is shown in Fig. S5. From the numerical simulations, it is therefore clear that the spectral coherence of the SC is very sensitive to the pump power fluctuation, which must be included to get a realistic value of the SC spectral coherence.

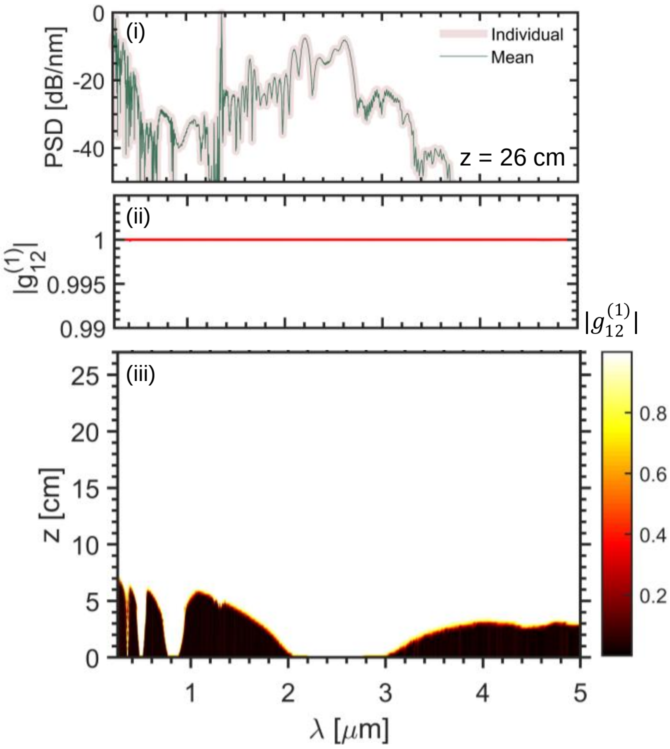

Fig. S4. (i) Power spectral density (PSD) at fiber length z = 26 cm. (ii) Modulus of the complex first order degree of spectral coherence of the generated SC, $\left|g_{12}^{(1)}\right|$, at z = 26 cm. (iii) Evolution of $\left|g_{12}^{(1)}\right|$ along the fiber. The coherence properties were found by averaging over 100 simulations with only OPPM noise, i.e., ignoring pump laser noise.

The relative intensity noise (RIN) was calculated as the ratio between the standard deviation σ(ω) and the mean μ(ω) [13]

$$RIN(\omega) = \frac{\sigma(\omega)}{\mu(\omega)} = \frac{\sqrt{< \left(|(A_m)\,(\omega)|^2 - \mu(\omega)\right)^2 >}}{\mu(\omega)} \qquad (\boldsymbol{S9})$$

where μ(ω)= <|A_m(ω)|^2> The calculated RIN is shown in Fig. S6. It can be seen from Fig. S6 that the RIN is very low in the full spectrum when pump noise fluctuation is ignored, whereas it increases drastically when pump noise fluctuation in added.

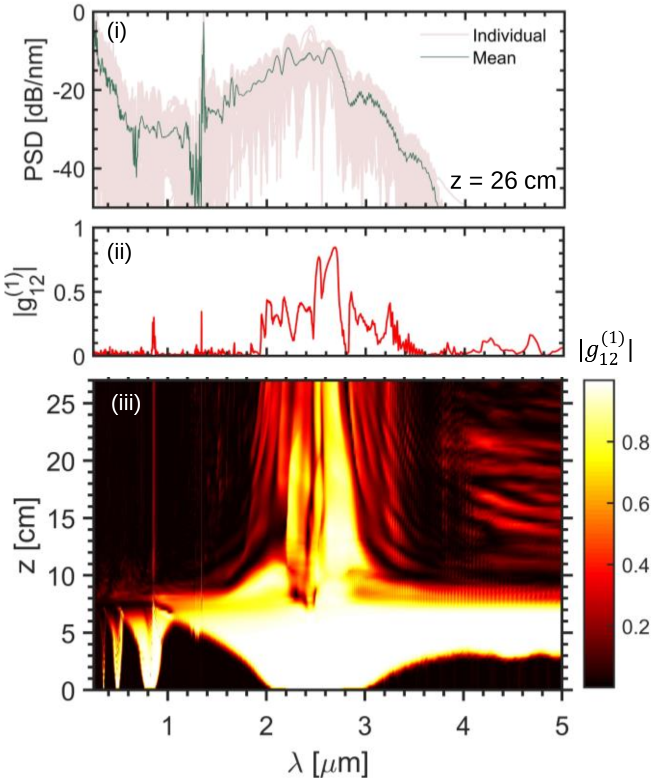

Fig. S5. (i) PSD at fiber length z = 26 cm. (ii) Modulus of the complex first order degree of spectral coherence of the generated SC, $\left|g_{12}^{(1)}\right|$, at z = 26 cm. (iii) Evolution of $\left|g_{12}^{(1)}\right|$ along the fiber. The coherence properties were found by averaging over 100 simulations with both OPPM noise and a pump laser noise of 5.5%. The strongly reduced spectral coherence clearly demonstrates the significant effect of the pump noise on the RIN of the SC.

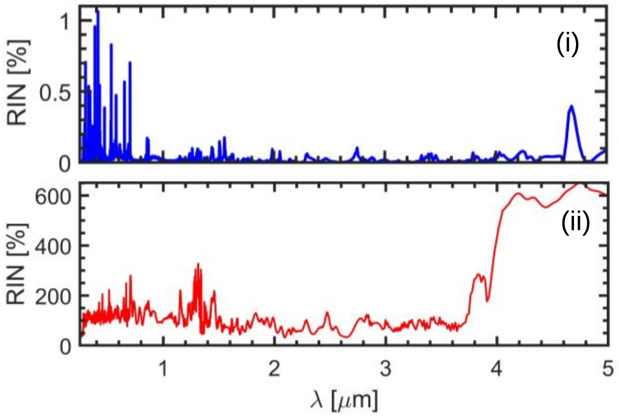

Fig. S6. Calculated RIN with 100 realizations for fiber length z = 26 cm. (i) Without pump noise fluctuations, and (ii) with 5.5% pump laser fluctuations.

REFERENCES

1. Maria, M. *et al.* Q-switch-pumped supercontinuum for ultra-high resolution optical coherence tomography. *Opt. Lett., OL* **42**, 4744–4747 (2017).
2. Govind Agrawal. Nonlinear Fiber Optics - 5th Edition. https://www.elsevier.com/books/nonlinear-fiber-optics/agrawal/978-0-12-397023-7 (2012).
3. Frosz, M. H., Falk, P. & Bang, O. The role of the second zero-dispersion wavelength in generation of supercontinua and bright-bright soliton-pairs across the zero-dispersion wavelength. *Opt. Express, OE* **13**, 6181–6192 (2005).
4. Satsuma, J. & Yajima, N. B. Initial Value Problems of One-Dimensional Self-Modulation of Nonlinear Waves in Dispersive Media. *Prog Theor Phys* **55**, 284–306 (1974).
5. Mak, K. F., Travers, J. C., Hölzer, P., Joly, N. Y. & Russell, P. S. J. Tunable vacuum-UV to visible ultrafast pulse source based on gas-filled Kagome-PCF. *Opt. Express, OE* **21**, 10942–10953 (2013).
6. Joly, N. Y. *et al.* Bright spatially coherent wavelength-tunable deep-UV laser source using an Ar-filled photonic crystal fiber. *Phys. Rev. Lett.* **106**, 203901 (2011).
7. Chen, C.-M. & Kelley, P. L. Nonlinear pulse compression in optical fibers: scaling laws and numerical analysis. *J. Opt. Soc. Am. B, JOSAB* **19**, 1961–1967 (2002).
8. Novoa, D., Cassataro, M., Travers, J. C. & Russell, P. S. J. Photoionization-Induced Emission of Tunable Few-Cycle Midinfrared Dispersive Waves in Gas-Filled Hollow-Core Photonic Crystal Fibers. *Phys. Rev. Lett.* **115**, 033901 (2015).
9. Travers, J. C., Chang, W., Nold, J., Joly, N. Y. & Russell, P. S. J. Ultrafast nonlinear optics in gas-filled hollow-core photonic crystal fibers [Invited]. *J. Opt. Soc. Am. B, JOSAB* **28**, A11–A26 (2011).
10. Hasan, M. I., Akhmediev, N. & Chang, W. Empirical Formulae for Dispersion and Effective Mode Area in Hollow-Core Antiresonant Fibers. *J. Lightwave Technol., JLT* **36**, 4060–4065 (2018).
11. Ermolov, A., Mak, K. F., Frosz, M. H., Travers, J. C. & Russell, P. St. J. Supercontinuum generation in the vacuum ultraviolet through dispersive-wave and soliton-plasma interaction in a noble-gas-filled hollow-core photonic crystal fiber. *Phys. Rev. A* **92**, 033821 (2015).
12. Dudley, J. M., Genty, G. & Coen, S. Supercontinuum generation in photonic crystal fiber. *Rev. Mod. Phys.* **78**, 1135–1184 (2006).
13. Gonzalo, I. B., Engelsholm, R. D., Sørensen, M. P. & Bang, O. Polarization noise places severe constraints on coherence of all-normal dispersion femtosecond supercontinuum generation. *Scientific Reports* **8**, 6579 (2018).
